# Copper homeostasis and cuproptosis in tumor biology: mechanistic insights and clinical perspectives

**DOI:** 10.3389/fimmu.2026.1908734

**Published:** 2026-08-17

**Authors:** Zian Wang, Jiahao Xie, Yu Han, Xiaotao Zhang, Jinyu Song, Yu Xing, Binbin Hou, Cong Han, Yi Sui

**Affiliations:** 1 Department of Gastrointestinal Surgery, The Second Hospital of Dalian Medical University, Dalian, Liaoning, China; 2 Department of Dermatology, The Second Hospital of Dalian Medical University, Dalian, Liaoning, China; 3 Intensive Care Unit Department, The Second Hospital of Dalian Medical University, Dalian, Liaoning, China

**Keywords:** biomarkers, clinical translation, copper homeostasis, cuproptosis, nanotherapeutics, regulated cell death, tumor biology, tumor immunity

## Abstract

Copper is considered a necessary trace element that plays crucial roles in energy production in mitochondria, redox regulation, and metabolism. Copper homeostasis disturbance may promote tumor initiation and progression and therapy resistance. Cuproptosis is a regulated form of cell death when mitochondrial copper interacts with the proteins from the citric acid cycle causing their oligomerization and aggregation, loss of Fe–S proteins, proteotoxic stress, and cell death. Unlike apoptosis, ferroptosis, and necroptosis, cuproptosis can be promoted in cells keeping functioning lipoylation of proteins and actively using mitochondrial oxidative metabolism. Copper also influences tumor-related signaling pathways, induces angiogenesis, invasion, metabolic rewiring, and tumor immunity. This review summarizes copper homeostasis and cuproptosis, possible biomarkers, and connections between cuproptosis and other types of regulated cell death. In addition, it concerns copper chelators, ionophores, targeted nanotherapy with high copper content, the combination of copper therapy with chemotherapy, radiotherapy, and immunotherapy and it presents recent clinical data on the therapeutic effect of copper agents.

## 1 Introduction

Modern copper biology emerged through a sequence of nutritional, human-genetic, and model-organism discoveries. In 1928, Hart et al. showed that copper supplementation supported effective hemoglobin formation in rats, establishing the biological essentiality of copper in mammalian nutrition (1). Human genetic disorders subsequently revealed the consequences of disrupted copper transport: Menkes disease, which produces systemic functional copper deficiency, was traced to defects in ATP7A, whereas Wilson disease, characterized by pathological copper accumulation, was traced to defects in ATP7B (2, 3). Positional cloning of these disease genes in 1993 identified homologous copper-transporting P-type ATPases and provided a molecular entry point into mammalian copper trafficking (2, 3). In parallel, yeast genetics identified CTR1 as a high-affinity copper-uptake gene (4), and complementation of a Saccharomyces cerevisiae ctr1 mutant subsequently led to the isolation of human CTR1/SLC31A1 (5). In 2022, Tsvetkov et al. mechanistically defined cuproptosis as a distinct form of copper-dependent regulated cell death (6). Together, these milestones established the uptake–distribution–export framework that now underpins studies of copper homeostasis, tumor copper dependence, and cuproptosis.

**Figure 1:**
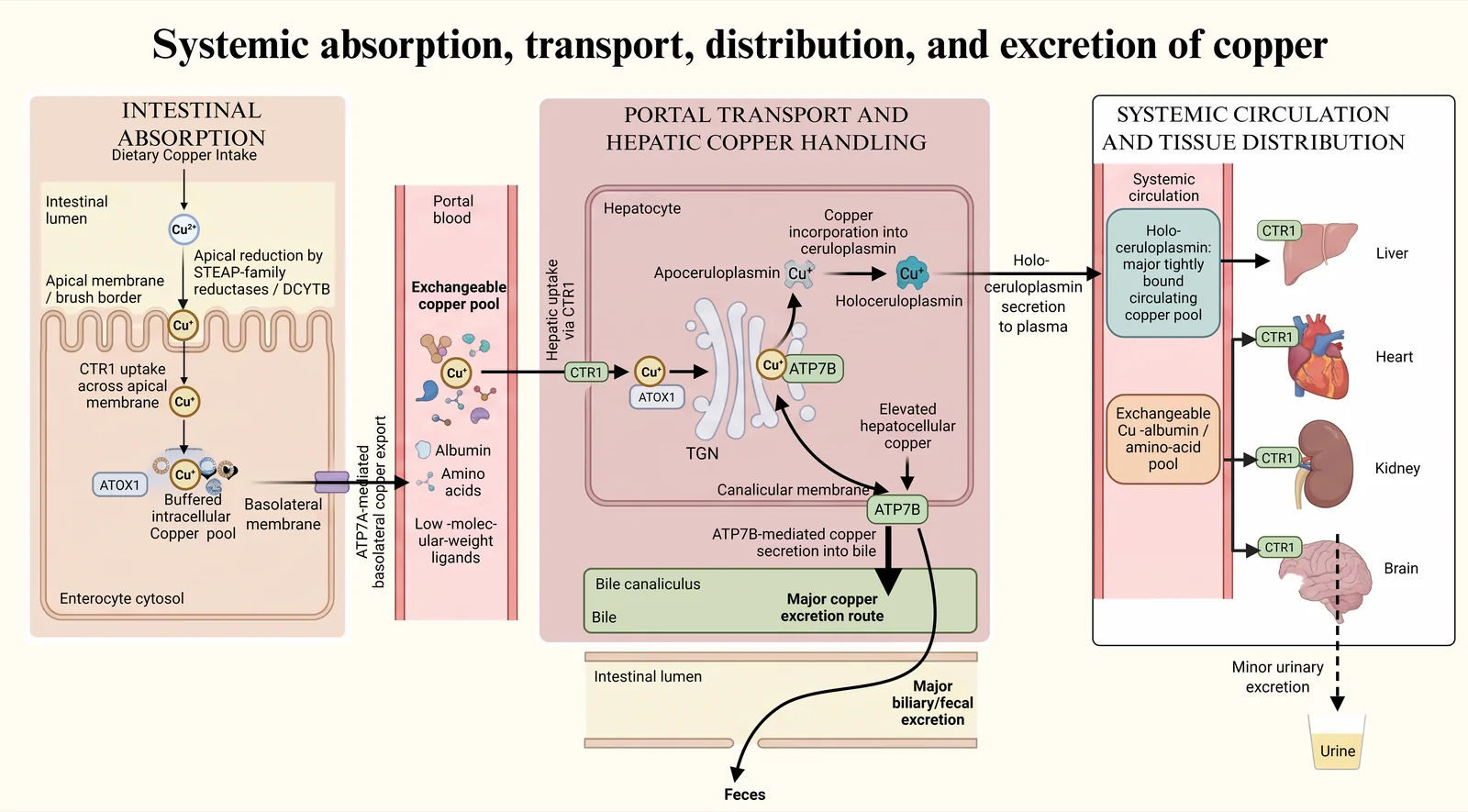
Systemic absorption, transport, distribution, and excretion of copper. Dietary Cu(II) is reduced to Cu(I) at the apical surface of enterocytes and subsequently imported through copper transporter 1 (CTR1). ATP7A transports copper across the basolateral membrane into the portal circulation. In hepatocytes, ATP7B delivers copper to the trans-Golgi network for incorporation into apoceruloplasmin and, under copper-overload conditions, redistributes toward canalicular compartments to promote biliary copper excretion (7–9). In the circulation, copper is transported mainly as ceruloplasmin-bound copper and, to a lesser extent, as an exchangeable pool associated with albumin, amino acids, and other low-molecular-weight ligands. Copper is distributed to peripheral tissues, whereas urinary copper excretion is normally minor. The image was created by the authors using BioRender.

Copper is widely present in various types of human cells. As a cofactor of multiple metalloenzymes, it not only participates in cellular metabolism but also plays a key role in multiple physiological processes, including antioxidant defense, neurotransmitter synthesis and regulation, immune regulation, connective-tissue maturation, and angiogenesis (7–11). Because unbound copper can participate in redox reactions and damage cellular macromolecules, its uptake, intracellular trafficking, storage, and export are tightly controlled. Copper is mainly absorbed in the small intestinal tract and circulates in exchangeable forms bound to albumin, amino acids, and other low-molecular-weight ligands, while ceruloplasmin (Cp) carries most circulating copper. CTR1 mediates high-affinity copper uptake, whereas copper chaperone for superoxide dismutase (CCS), cytochrome c oxidase copper chaperone 17 (COX17), and antioxidant 1 (ATOX1) distribute copper to SOD1, mitochondrial cytochrome c oxidase, and ATP7A/ATP7B, respectively (7–9, 11–13). Copper shows its highest tissue concentration in the liver, with lower levels in the brain, heart, and kidneys. Excess copper is primarily eliminated through biliary secretion into the intestine. As a trace element, copper homeostasis must be closely controlled, because copper deficiency or excess can lead to inherited, metabolic, neurodegenerative, cardiovascular, and malignant diseases (8, 9, 14–17). Detailed information on how cells take up, transport, store, and excrete copper is required for understanding copper-homeostasis regulation and for developing therapies that target it (**Figure 1**).

According to earlier reports, copper serves an important function both as a fundamental trace element and as a component that influences tumorigenesis and tumor progression. Tumor cells modify copper usage to support proliferation, migration, angiogenesis, autophagy, metabolic remodeling, and environmental adaptation. At the biochemical level, copper is capable of activating PDPK1-AKT signaling, BRAF-MEK-ERK pathway, ULK1/2-dependent autophagy, and extracellular matrix remodeling (18–25). Changes in the genes encoding proteins such as CTR1, ATOX1, ATP7A, ATP7B, and CTR2 may influence tumor growth and metastasis, platinum accumulation, and treatment response (12, 24, 26–31). Hence, copper acts as a double-edged sword: on the one hand, a certain level of copper is necessary for normal processes in cells, whereas too much copper found in mitochondria can lead to cell death (6, 15, 25, 26, 32).

The increased quantities of copper react with the lipoylated mitochondrial proteins of the tricarboxylic acid (TCA) cycle, especially with DLAT, causing unusual oligomerization and aggregation (6, 26, 33). The mentioned changes are accompanied by the loss of Fe-S-cluster proteins and the occurrence of proteotoxic stress. FDX1 contributes to the process by reducing Cu(II) bound to elesclomol (ES), thereby facilitating the release of copper from the ionophore within mitochondria and supporting LIAS-dependent protein lipoylation (34–36). Hence, the cells that have working lipoylation system and rely on oxidative phosphorylation (OXPHOS) will react more readily to cuproptosis as compared to the cells that rely mostly on glycolysis (6, 34, 35, 37).

Copper’s value lies not only in its basic tumor-killing mechanisms, but also in its possible role in affecting programmed death ligand 1 (PD-L1) regulatory processes and immune responses (38, 39). Concomitantly with such copper depletion caused by tetrathiomolybdate (TM) therapy, myeloid-derived suppressor cell activity may decrease, collagen deposition may regress, and CD4+ T-cell infiltration may significantly escalate in preclinical models of triple-negative breast cancer (TNBC) (40). The results suggest that cuproptosis allows tumor cells to release damage-associated molecular patterns (DAMPs) and activate dendritic cells and that T-cell-mediated interferon-γ (IFN-γ) generation may also constitute a means to upregulate FDX1 gene expression and increase sensitivity to cuproptosis (41). Such findings thus provide a rationale for the use of immune checkpoint blockade in combination with copper-based therapies and highlight the context-dependent character of immune responses.

Several points are now well established: cellular copper homeostasis depends on coordinated uptake, buffering and chaperoning, organellar delivery, and ATP7A/ATP7B-mediated redistribution or export; canonical cuproptosis additionally requires mitochondrial copper loading, intact protein lipoylation, and active TCA-cycle/OXPHOS metabolism (6–9, 34–36). Major questions remain regarding how spatially distinct copper pools are remodeled across tumor types, which biomarkers can distinguish canonical cuproptosis from other forms of copper-induced cytotoxicity in patients, and how copper depletion or overload can be delivered selectively without disrupting essential systemic copper physiology (26, 42–47). Building on recent comprehensive reviews (42–46), this article integrates the historical genetics and cell biology of copper transport with tumor signaling, the molecular definition of cuproptosis, candidate biomarkers, resistance mechanisms, therapeutic strategies, and available clinical evidence. Particular emphasis is placed on separating established mechanisms from emerging or unvalidated claims and on defining priorities for biomarker-guided clinical translation. Key candidate biomarkers, comparisons with other regulated cell-death modalities, representative preclinical nanotherapeutic strategies, and selected clinical studies are summarized in **Tables 1**–**4**.

**Table 1:** Candidate copper-related and cuproptosis-associated biomarkers in cancer.

Biomarker	Specimen	Biological rationale	Potential use	Key limitations
Total copper/ceruloplasmin	Serum or plasma	Reflects systemic copper status and acute-phase response	Exploratory diagnostic or prognostic marker (85–89)	Nonspecific; affected by inflammation, liver function, nutrition, and assay method
Copper-to-zinc ratio	Serum	Integrates copper elevation and zinc reduction	Prognostic enrichment in breast cancer (86, 88)	Requires external validation and standardized cutoffs
Exchangeable or labile copper	Plasma, tumor, or imaging probe	Closer to bioavailable copper than total copper	Pharmacodynamic monitoring and target engagement	Assays are not standardized for oncology
CTR1/SLC31A1	Tumor tissue	Copper and platinum uptake	Select copper-dependent tumors; predict platinum uptake (27, 28)	Expression does not directly measure transport activity
ATP7A/ATP7B	Tumor tissue	Copper and platinum export or sequestration	Resistance biomarker for copper overload and platinum therapy (27, 29, 30)	Subcellular localization and trafficking matter
FDX1, LIAS, LIPT1, lipoylated DLAT	Tumor tissue	Defines a permissive lipoylation state for cuproptosis	Patient selection and pharmacodynamic confirmation (6, 35, 36)	No clinically validated assay or threshold
DLAT oligomerization and Fe-S-protein loss	Paired tumor biopsies	Proximal molecular evidence of canonical cuproptosis	On-treatment proof of mechanism (6)	Requires fresh or optimized tissue assays
OXPHOS/TCA-cycle signature	Tumor tissue, functional imaging, ex vivo assay	Indicates dependence on mitochondrial respiration	Select tumors vulnerable to copper depletion or overload (6, 51, 52)	Metabolic state is spatially and temporally heterogeneous
Metallothioneins and GSH pathway	Tumor tissue	Copper sequestration and buffering	Predict resistance to cuproptosis (49, 51)	Redundant antioxidant and metal-buffering mechanisms

**Table 2:** Comparison of cuproptosis with major regulated cell-death modalities.

Modality	Core trigger/dependency	Execution features	Representative markers	Typical rescue or validation
Cuproptosis	Mitochondrial copper, protein lipoylation, OXPHOS (6)	Lipoylated-protein aggregation, Fe-S-protein loss, proteotoxic stress	FDX1/LIAS/LIPT1 dependence; lipoylated-DLAT aggregation	Copper chelation; genetic disruption of lipoylation; reduced respiration
Apoptosis	Death receptors or mitochondrial outer-membrane permeabilization (53)	Caspase cascade; nuclear fragmentation; membrane initially preserved	Cleaved caspase-3/7, PARP cleavage, cytochrome-c release	Caspase inhibition; BCL-2-family manipulation
Ferroptosis	Iron-dependent phospholipid peroxidation (99)	Catastrophic membrane lipid oxidation	Lipid ROS, GPX4 loss, ACSL4 dependence	Ferrostatin-1/liproxstatin-1; iron chelation
Necroptosis	RIPK1/RIPK3 signaling (53, 100)	MLKL oligomerization and membrane rupture	Phosphorylated RIPK3 and MLKL	RIPK1/RIPK3/MLKL inhibition or knockout
Pyroptosis	Inflammatory caspases or selected caspase-gasdermin pathways (101)	Gasdermin pores, cell swelling, inflammatory mediator release	Cleaved GSDMD/GSDME, IL-1β/IL-18 release	Gasdermin or inflammatory-caspase inhibition

**Table 3:** Representative preclinical copper-based nanotherapeutic strategies.

Platform	Delivery concept	Reported mechanism	Combination/immune effect	Translational limitation
Stimulus-responsive Cu(I) complex nanoparticles (118)	ROS-responsive tumor release	DLAT aggregation and cuproptosis	Immunogenic response in pancreatic-cancer models	Preclinical; biodistribution and chronic copper retention require study
Celastrol-Cu nanoparticles (119)	Copper ionophore plus GSH scavenging	Self-amplified cuproptosis	Enhanced checkpoint-blockade response in mice	Celastrol and copper toxicology; manufacturing reproducibility
Elesclomol-loaded CuO nanoplatform (120)	Co-delivery of ionophore and copper source	Mitochondrial copper overload and cuproptosis	Enhanced antitumor immunity in animal models	No human pharmacokinetic or safety data
ROS-responsive NP@ESCu (121)	Triggered co-release of elesclomol and copper	Cuproptosis with tumor-microenvironment remodeling	Combined with anti-PD-L1	Passive tumor accumulation may not translate
Copper-chlorogenic-acid nanoparticles (102)	Metal-phenolic self-assembly	Cuproptosis plus pyroptosis and redox disruption	DAMP release, dendritic-cell activation, CD8+ T cells	Mixed death mechanisms; preclinical only

**Table 4:** Selected completed and ongoing clinical studies of copper-modulating therapies in cancer.

Intervention	Cancer/design	Status and size	Main finding	Interpretation
Elesclomol + paclitaxel (58)	Stage IV melanoma; randomized phase III	Terminated; 651 randomized in the publication (registry enrollment: 630); 651 in publication	No significant PFS benefit overall; death imbalance concentrated in high-LDH patients; normal-LDH subgroup signal	Supports metabolic selection; not an approved cuproptosis therapy
Disulfiram + copper + chemotherapy (106)	Recurrent glioblastoma; randomized phase II/III	Completed; 88	No survival benefit; more grade ≥3 and serious adverse events	Unfavorable risk-benefit in unselected recurrent GBM
Disulfiram + copper + temozolomide (107)	Recurrent TMZ-resistant GBM; single-arm phase II	Completed; 23	ORR 0%; median PFS 1.7 months; median OS 7.1 months	Limited activity despite tolerability
DSF/Cu + RT/TMZ, NCT02715609 (108)	Newly diagnosed GBM; phase I/II	Completed; 35; registry results posted	Designed for pharmacokinetics, safety, and efficacy	Results require interpretation with published data and mechanism confirmation
DSF/Cu + TMZ, NCT03363659 (109)	Unmethylated GBM; phase II	Terminated; 15	Registry results available	Small single-arm study; not definitive
Tetrathiomolybdate (71)	High-risk breast cancer; phase II	Completed; 40	Copper depletion and EPC reduction feasible; hematologic toxicity observed	Pharmacodynamic activity, not definitive survival proof
Penicillamine + low-copper diet (122)	Newly diagnosed GBM; phase II vs historical controls	Completed; 40	Hypocupremia achieved; no survival improvement	Demonstrates feasibility but not efficacy
TM + capecitabine +/- pembrolizumab, NCT06134375 (123)	High-risk residual TNBC; phase 1b/randomized phase 2	Recruiting; estimated 204; efficacy and safety outcomes pending	Safety and recurrence outcomes pending	Important prospective test of combination and patient selection

## 2 Mechanisms of intracellular copper homeostasis

Copper is an essential cofactor for numerous enzymes, such as cytochrome c oxidase, superoxide dismutase (SOD), and lysyl oxidase (LOX). As a result, it makes an important contribution to redox equilibrium, antioxidant protection, iron metabolism, production of neurotransmitters, angiogenesis, and immune regulation. Despite the very small amount of copper found in the body, both deficiency and excess can result in serious diseases, which means that copper homeostasis is vital for health. The source of copper is the food that a person takes; animal liver, shellfish, nuts, and whole grain products are the main sources of copper (7–9, 48). Copper absorption mainly occurs in the duodenum and proximal jejunum. At the apical membrane, Cu(II) is reduced to Cu(I) caused by metalloreductases, which include STEAP-proteins and duodenal cytochrome b; this is followed by high-affinity uptake through CTR1 proteins. Then there is a process of copper transport across the basolateral membrane and it is sent to the hepatic portal vein due to the activity of ATP7A transporting copper. Bringing copper to the liver is carried out by albumin, amino acids, and other low-molecular-weight ligands that take copper from the blood to the liver. From the liver copper is provided to the TGN by ATP7B to be incorporated into Cp, then canalicular vesicles are supplied with copper (7–9). The major route of copper elimination is bile; urinary excretion of copper is insignificant (8, 9) (**Figure 1**).

### 2.1 Uptake and transport of copper in cells

There is a low concentration of free copper in the body to protect against oxidative damage because free copper generally binds to its chaperone proteins and is buffered by an antioxidant such as metallothionein and glutathione (GSH) (7–9, 12, 13, 49). There are three main classes of copper chaperone protein; they are CCS, COX17, and ATOX1. CCS carries copper to copper-zinc-having superoxide dismutase enzyme known as “SOD1”. SOD1, which is located mainly in the cytosol and the mitochondrial intermembrane space, promotes the conversion of superoxide to oxygen and hydrogen peroxide; thereby limiting superoxide-mediated oxidative damage and maintaining redox homeostasis. COX17 is synthesized in the cytoplasm and is directed to the mitochondrial intermembrane space; on reaching COX17, it works with COX11, SCO1, SCO2, COA4, COA6, delivering copper to cytochrome c oxidase (9, 11–13). Overall, these processes lead to antioxidant protection, mitochondrial respiration, functioning of cuproenzymes in secretory pathway, and copper export from cells.

### 2.2 Storage and excretion of copper in cells

**Figure 2:**
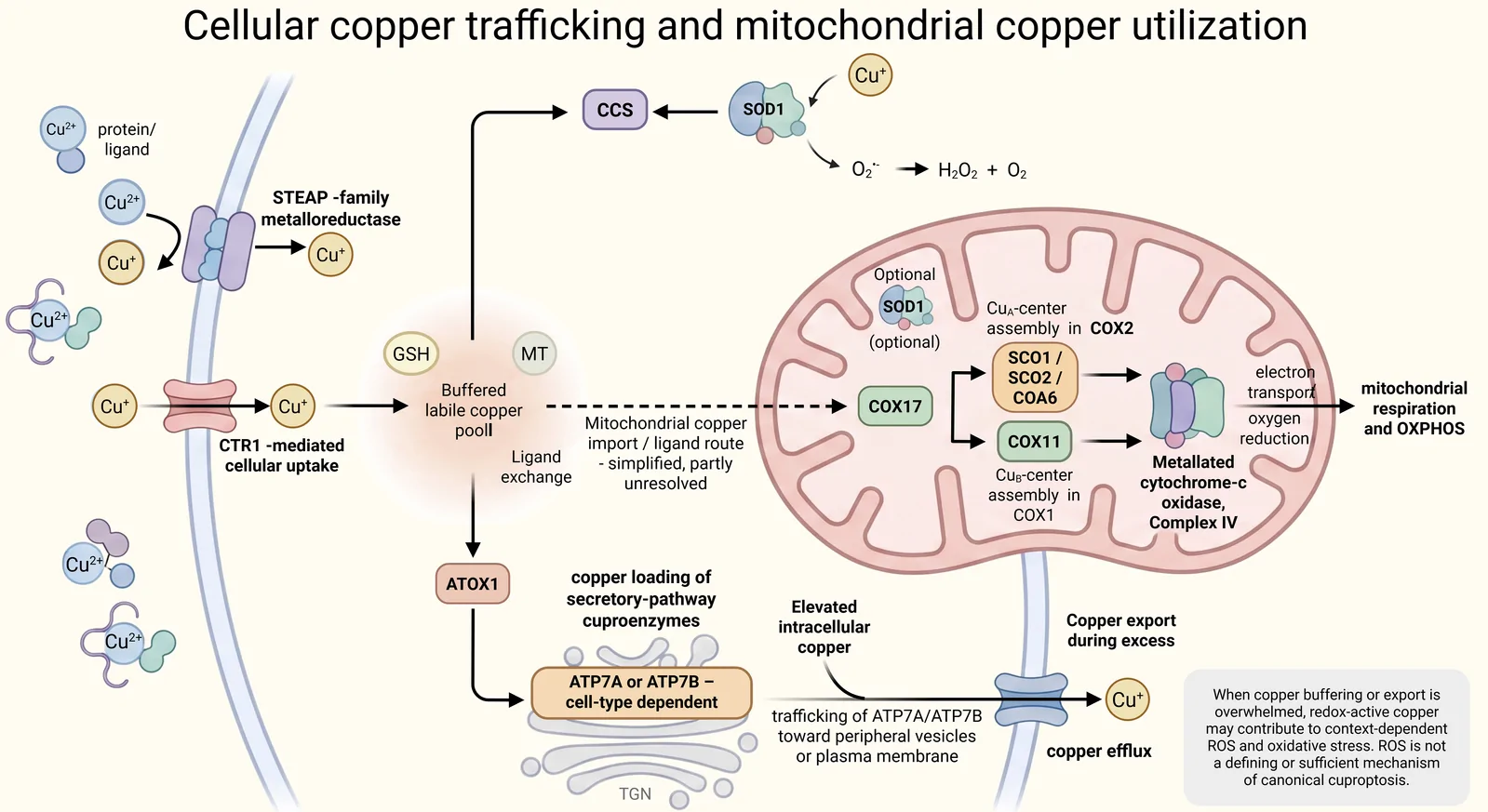
Cellular copper trafficking and mitochondrial copper utilization. Extracellular Cu(II) is reduced to Cu(I) before high-affinity cellular uptake through CTR1. Intracellular Cu(I) is maintained within a buffered labile pool by glutathione and metallothioneins and is transferred through specialized copper chaperones. CCS delivers copper to SOD1, COX17 participates in mitochondrial cytochrome c oxidase assembly, and ATOX1 transfers copper to ATP7A or ATP7B in the trans-Golgi network (7–9, 11–13). When intracellular copper levels increase, ATP7A and ATP7B redistribute toward vesicular or plasma-membrane-associated compartments to promote copper efflux (1–3, 43). Impaired copper buffering or export may contribute to reactive oxygen species generation and oxidative stress; however, ROS production alone is insufficient to define canonical cuproptosis (6, 49, 50).

ATOX1, as another important intracellular copper chaperone, is primarily localized to the cytosol and nucleus and plays a key role in intracellular copper trafficking and efflux (7–9). Its high-affinity binding to Cu(I) limits the pool of free copper, establishing a dynamic intracellular buffering system that helps prevent copper-induced oxidative stress. The ATOX1-Cu(I) complex targets and transports copper ions to the trans-Golgi network (TGN), where it transfers copper to ATP7A or ATP7B. When intracellular copper levels rise, ATP7A or ATP7B redistribute from the TGN to vesicular or plasma-membrane-associated compartments and promote copper export (6, 49). Metallothioneins and GSH provide additional buffering, whereas GSH depletion or impaired copper export can lower the threshold for copper-induced cytotoxicity. The abundance and localization of these proteins are therefore plausible determinants of cuproptosis sensitivity and resistance (**Figure 2**).

### 2.3 Tumor-specific remodeling of copper metabolism

Several tumors reflect increasing demand for copper, but both direction and extent of copper remodeling depend on histology, stage, anatomical site and treatment history. Protein CTR1 being overexpressed favors copper influx and facilitates copper-mediated signaling, while proteins ATP7A and ATP7B support copper efflux and change the sensitivity to copper overload and cisplatin drugs (25–30). Additionally, ATOX1 helps to transport copper in the direction of secretory cuproenzymes, such as members of lysyl oxidase family (12, 24). Tumor hypoxia brings about one more degree of regulation: hypoxia-inducible factor 1α (HIF-1α) helps induce pyruvate dehydrogenase kinases 1 and 3 (PDK1/3) and decreases quantity of lipoylated DLAT Targets, therefore increasing copper sequestering by metallothionein and contributing to the resistance to cuproptosis (51).

Metabolic heterogeneity is quite crucial. Tumor populations that function with intense OXPHOS activities could either need more amounts of mitochondrial copper or might have increased predisposition to either copper deficiency or excess based on the treatment (6, 51, 52). An example can be the case study of the TNBC model wherein mitochondrial complex IV was impaired from lack of copper that resulted in OXPHOS diminishment and inhibition of the metastasis in a particular tumor cell subpopulation (52). Thus, it becomes apparent that there isn’t a universal method: in one and the same copper-modulating agent may work well in an OXPHOS-dependent cancer while being harmful for a hypoxic or glycolytic or copper-export-ready one.

## 3 The molecular basis of cuproptosis

Mitochondria function as integral centers of cellular energy metabolism and also take part in different types of regulated cell death (11, 32, 33, 53). Cuproptosis is dependent on mitochondrial respiration while being mechanistically distinct from apoptosis, ferroptosis, necroptosis, and pyroptosis. The distinct characteristics of cuproptosis include protein-lipoylation dependence, copper-induced oligomerization, loss of Fe-S-cluster proteins, and proteotoxic stress (6, 26). Cuproptosis cannot happen with only copper accumulation.

### 3.1 FDX1 as a redox and lipoylation gatekeeper

**Figure 3:**
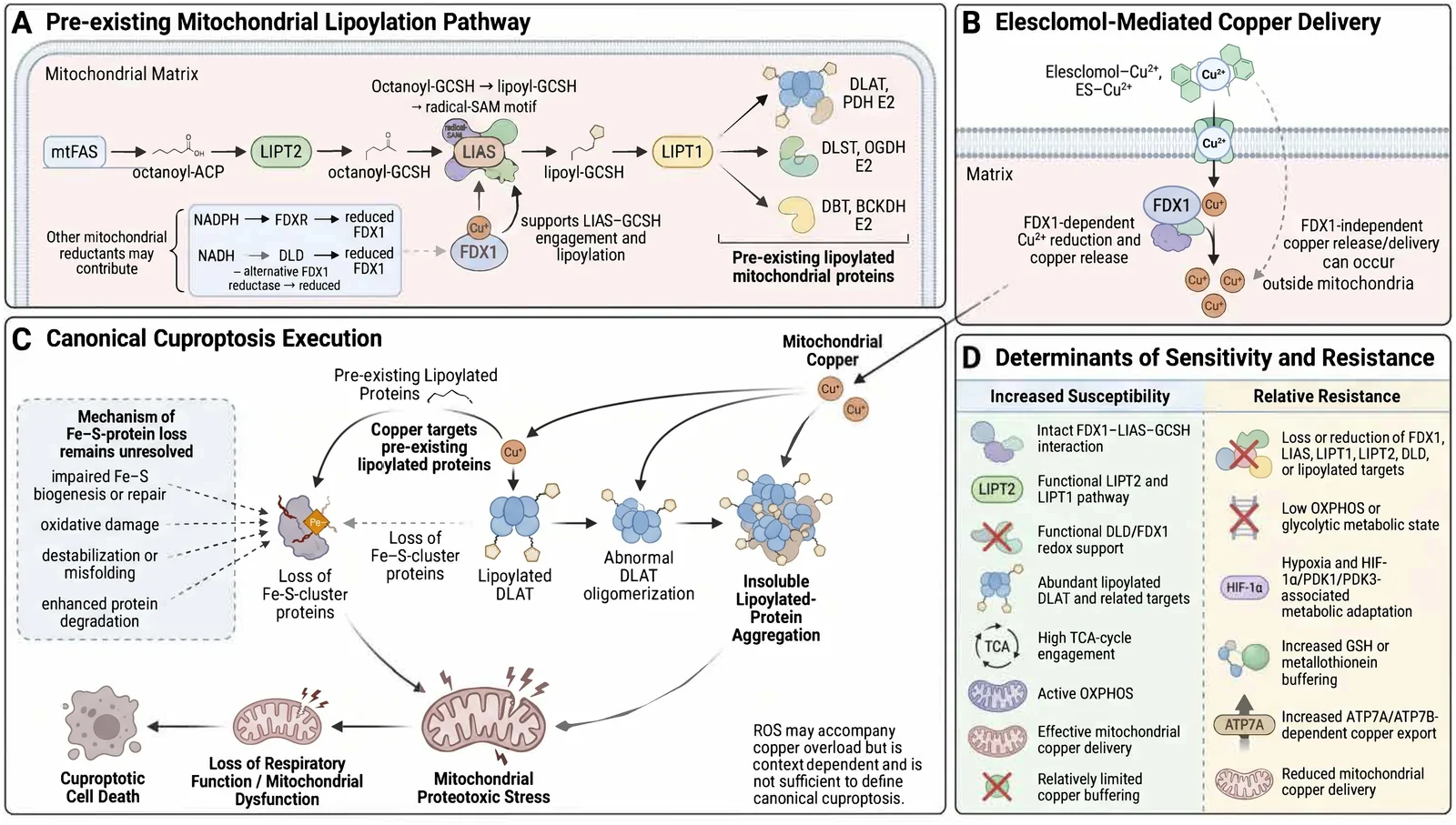
Core molecular determinants of cuproptosis. **(A)** Pre-existing mitochondrial lipoylation pathway. FDX1 supports mitochondrial protein lipoylation through its interaction with lipoic acid synthase (LIAS) (35, 36), while LIAS and LIPT1 generate lipoylated mitochondrial enzyme targets, including DLAT and DLST. **(B)** Elesclomol-mediated copper delivery. Elesclomol transports Cu(II); within mitochondria, FDX1 can reduce ionophore-bound Cu(II) to Cu(I), facilitating mitochondrial copper release, whereas FDX1-independent elesclomol-mediated copper release can occur outside mitochondria (34). **(C)** Canonical cuproptosis execution. Excess mitochondrial copper binds to pre-existing lipoylated proteins, particularly lipoylated DLAT, and promotes their abnormal oligomerization and aggregation. These events are accompanied by loss of iron-sulfur proteins, proteotoxic stress, mitochondrial dysfunction, and cell death (6). **(D)** Determinants of susceptibility and resistance. Intact mitochondrial protein lipoylation and active TCA-cycle/OXPHOS metabolism increase susceptibility to cuproptosis, whereas impaired lipoylation, glycolytic adaptation, copper sequestration, and enhanced copper export promote resistance (6, 49, 51). The image was created by the authors using BioRender.

FDX1 is a mitochondrial [2Fe-2S] ferredoxin that plays a role in cuproptosis induction (6, 34–36, 54). It is not a membrane-associated copper receptor, but rather functions as a redox-metabolic gate in mitochondrial copper chemistry and lipoylated protein synthesis process. During the copper transfer through ES, reduced FDX1 converts Cu(II) bound by ES to Cu(I) thus lowering the affinity of copper to the ionophore and supporting copper release in the mitochondria. However, the release of copper may occur under other intracellular circumstances irrespective of the role of FDX1, as the expression of FDX1 is important but not the only factor affecting the activity of ES (34) (**Figure 3B**).

FDX1 additionally promotes mitochondrial protein lipoylation. Biochemical experiments indicate that FDX1 interacts with lipoic acid synthase (LIAS), thus promoting the interaction of LIAS with its partner protein glycine cleavage system protein H (GCSH) (35). Loss of FDX1 decreases lipoylated mitochondrial proteins, which causes disruption in respiration and decreases sensitivity to cuproptosis (6, 35). Synthesis of structure-function relationships revealed the shared determinants of FDX1 working for both lipoylation and cuproptosis (36). Therefore, the definition of ‘FDX1-mediated copper sensing’ should be considered as redox-metabolic gating. Specifically, FDX1 contributes both to copper release from the ionophore and to the availability of lipoylated targets, which makes it a key player in this process.

Regulation regarding the concentration of FDX1 is also dependent on the specific context. In steroidogenic cells, SF1 and c-JUN work together at the promoter of FDX1 (55). The lactylation of METTL16 in gastric cancer has been shown to increase FDX1 mRNA stability in relation to m6A regulatory processes (56). A specific miR-21-5p/FDX1 relationship has also been described in clear-cell renal carcinoma (57). Observations of this nature suggest that the regulation of FDX1 is tumor-type specific; however, it should be noted that these findings do not imply that one common upstream regulator exists for all cancer types (**Figure 3A**).

### 3.2 The lipoylation pathway and TCA-cycle dependency

DLAT is an essential element of mitochondrial pyruvate dehydrogenase complex E2 enzyme. When in the form of lipoate, DLAT takes part in the process of transforming pyruvate into acetyl-CoA and relates to glycolysis and TCA cycle in terms of energy metabolism (6, 35). More generally, lipoylation of proteins is needed in the case of different mitochondrial enzyme systems. LIAS inserts sulfur atoms into the octanoyl moiety on GCSH, while LIPT1 transfers the resulting lipoyl group from GCSH to target E2 proteins, including DLAT and DLST. The key components are DLAT and DLST (6). DLAT functions as the E2 acetyltransferase component of the pyruvate dehydrogenase complex rather than as the sole enzyme responsible for the overall reaction (**Figure 3A**).

Genetic loss of FDX1, LIAS, LIPT1, DLD or other elements responsible for mitochondrial lipoylation diminishes susceptibility to copper ionophores (6, 35). Conversely, conditions that amplify TCA cycle flux and OXPHOS broaden functionally engaged lipoylated targets and heighten cuproptosis vulnerability (6, 37). Disruption in electron transport or transition to glycolysis can lead to comparative resistance. TCA cycle and OXPHOS dependence, so have biological plausibility as stratification variables, although they are still considered exploratory measures rather than validated predictive biomarkers (6, 51, 58).

### 3.3 Lipoylated-protein aggregation, Fe-S-protein loss, and proteotoxic stress

If the concentration of copper in mitochondria exceeds that which the mitochondria can contain, copper will bind to mitochondrial lipoylated proteins. Lipoylated DLAT protein displays differences in its oligomerization and aggregation ability following exposure to a copper ionophore (6). These aggregates of proteins result in proteotoxicity and disruption of mitochondrial proteostasis (6, 37). In this regard, the copper-induced formation of aggregates has been associated with loss of Fe-S-containing proteins, one of the key features of cuproptosis (6). Fe-S proteins are involved in the processes of electron transfer, catalysis, and cluster biogenesis (54, 59, 60).

The exact cause of the loss of Fe-S-proteins is still somewhat unknown. It has been suggested that destabilizing the mature complexes, influencing the formation of Fe-S, causing oxidative damage, causing wrong folding of the proteins, and increasing their destruction are possible causes of Fe-S-proteins loss (6, 50, 59–61). Overload with copper can provide the formation of free radicals, disrupt mitochondrial membrane potential, and activate apoptosis or various oxidative stress pathways (50, 62, 63). However, ROS formation alone does not establish cuproptosis, and antioxidants do not consistently rescue canonical cuproptosis (6).

The susceptibility of Fe-S clusters to oxygen-dependent disassembly is well established in model proteins (64), but extrapolation from such systems to mammalian cuproptosis remains indirect. Direct measurements of Fe-S-cluster assembly, turnover, and degradation during cuproptosis are therefore still required (**Figure 3C**).

### 3.4 Operational definition and terminology

To avoid conceptual confusion, the term cuproptosis in the present review is applied only when copper dependence is complemented by evidence of mitochondrial-respiration and protein-lipoylation dependence along with molecular evidence such as lipoylated-DLAT oligomerization or aggregation (6). Genetic findings concerning FDX1, LIAS, LIPT1, DLD, or any other relevant lipoylated proteins serve as supporting evidence (6, 35, 36). The more general term copper-induced cytotoxicity is applied in cases when treatment mainly leads to oxidative injury, apoptosis, proteasome abnormality, endoplasmic-reticulum disturbance, NPL4-p97 disruption, ferroptosis, or combined death phenotypes, without demonstrating canonical cuproptosis (32, 50, 65).

Methodological appraisals of cuproptosis detection indicate that no single marker is adequate (47). Thus, robust attribution should incorporate copper dependence, pathway-specific rescue, mitochondrial respiration and lipoylating state, DLAT oligomerization and aggregation, Fe-S-protein modification, and a lack of competing cell death programs (47, 66, 67).

This difference is particularly crucial for copper and disulfiram (DSF) compounds and copper-based nano-materials. The CuET metabolite of DSF may bind to the p97/VCP adapter NPL4 and contribute to the accumulation of ubiquitinated proteins and the resulting proteotoxic stress (65). Although these effects may occur at the same time as mitochondrial toxicity due to copper, the mere aggregation of NPL4 does not cause cuproptosis. Thus, it is critical to establish the molecular basis for assertions made in the studies rather than relying solely on the copper dependency of the phenomenon in question (**Figure 3D**).

## 4 Roles of copper in tumor progression and cellular signaling

### 4.1 Copper-mediated regulation of tumor growth and the tumor microenvironment

**Figure 4:**
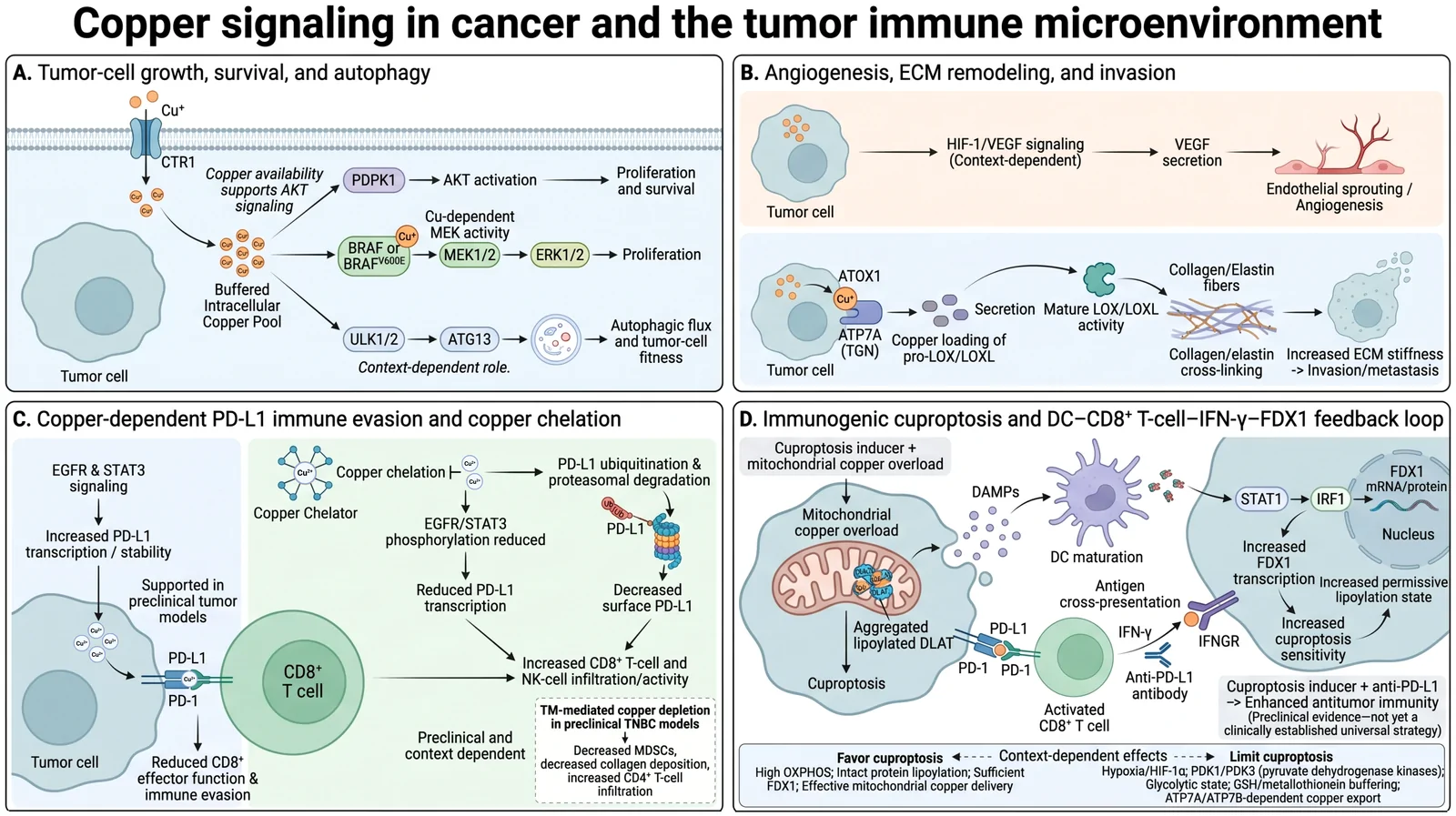
Copper signaling in cancer and the tumor immune microenvironment. **(A)** Physiological or elevated tumor copper supports cancer-cell proliferation, survival, and autophagy through CTR1-dependent PDK1–AKT signaling, BRAF–MEK–ERK signaling, and copper-dependent regulation of ULK1/2 (18–22, 24). **(B)** Copper promotes HIF-1α/VEGF-dependent angiogenesis and supports extracellular-matrix remodeling and invasion through ATOX1-, ATP7A-, and LOX/LOXL-dependent pathways (18, 20, 24). **(C)** Intratumoral copper promotes immune evasion by activating EGFR–STAT3 signaling and increasing PD-L1 expression, whereas copper chelation can promote PD-L1 degradation and enhance CD8+ T-cell and natural-killer-cell activity (39). **(D)** Therapeutic mitochondrial copper overload may induce cuproptosis and damage-associated molecular pattern release, thereby promoting dendritic-cell maturation, antigen presentation, and CD8+ T-cell activation. CD8+ T-cell-derived IFN-γ activates the STAT1–IRF1 axis, increases FDX1 transcription, and sensitizes tumor cells to cuproptosis, forming a positive feedback loop that may enhance anti-PD-L1 therapy (41). These effects remain context dependent and are influenced by hypoxia, OXPHOS dependence, copper buffering, copper export, and tumor type (49, 51). The image was created by the authors using BioRender.

Copper is of great importance for different processes of tumor cells’ growth, metabolism, angiogenesis, and autophagy. It serves as a promoter of the tumor growth-related pathways. Copper binding to PDK1, stimulates the interaction between PDK1 and AKT, activating AKT in a manner dependent on CTR1 (19). Additionally, copper serves as a necessary element for the oncogenic signaling pathway BRAF-MEK-ERK (22). In lung adenocarcinoma, it binds and controls ULK1/2. This shows the connection of the copper availability with autophagy as well as fitness of a tumor (21). Moreover, there are some dependencies of this on the certain signaling pathways and types of genotype (**Figure 4A**).

The pathways for growth and survival that respond to copper should also be understood in a wider context of metabolism controlled by FoxO, oxidative stress, and epigenetic changes (68, 69). The exposure to copper has shown to cause epigenetic and cytotoxic effects *in vitro* that have been studied on colon cancer cells (70), although dosages, chemicals used, and co-exposure sharply affect the results of this exposure.

Copper is an important regulator of both angiogenesis and autophagy. It promotes angiogenesis primarily through the induction of HIF-1-based transcription and copper-dependent extracellular-matrix enzymes (18, 20, 25). Copper depletion can decrease the production of angiogenic factors and circulating endothelial progenitor cells, but this process largely depends on the degree of copper deficiency and duration of this deficiency (71). HIF-1α may induce resistance to cuproptosis by decreasing lipoylated DLAT targets and enhancing copper sequestration via metallothioneins (51). Hence, hypoxia might help tumor adapt to the excess of copper (**Figure 4B**).

Signaling reliant on copper interacts with the nuclear processes and those linked to stress response. Studies employing ATOX1 proximity-labeling demonstrated that CRIP2 is a nuclear protein that binds copper and regulates autophagy activation (72), whereas prostate cancer research indicated that the direct targeting of ATOX1 leads to disruption of copper metabolism and induction of cuproptosis-like toxicity (73). These results expand the potential activities of ATOX1 beyond cytosolic copper transportation, although it is still not clear whether these activities are widely applicable to cancers.

### 4.2 Copper and tumor metastasis

Tumor metastasis is a major cause in cancer deaths and necessitates an integrated process of local invasion, intravasation, survival in circulation, extravasation, and colonization (74). As such, copper-mediated matrix remodeling is an important aspect but not the sole factor behind metastatic efficiency.

The enzyme LOX and its related proteins LOX-like are metal enzymes which require copper for the cross-linking of elastin and collagen, the raising of matrix stiffness and making possible the processes of invasion and forming metastatic niches (25, 26, 75–79). ATOX1 makes possible the directional distribution of copper and is necessary for breast cancer cells’ movement (12, 24). The lack of copper can result in collagen remodeling modification and a decrease of malignancy traits in preclinical and translational studies (40, 52). Overall, these results prove the copper-LOX/ATOX1 pathway to be regarded as a way for treatment despite the fact that the inhibition of this pathway may influence normal connective tissues and wound healing (**Figure 4B**).

### 4.3 Copper and tumor metabolism

The Warburg effect does not indicate that all cancers have the same type of glycolysis. There are many cancers that go through major mitochondrial respiration, and it is also possible that different metabolic groups contribute to the development of metastasis and resistance to treatment (6, 51, 52, 80). The activation of the OXPHOS pathway increases the amount of TCA-cycle proteins bearing a lipoyl group and increase susceptibility to cuproptosis. On the contrary, factors such as oxygen deficiency, PDK1/3 being activated, low levels of DLAT, induction of metallothionein, increased glutathione-mediated copper buffering, and its export can take this susceptibility away (49, 51). As a result, the expression of the FDX1 gene may not be by itself sufficient for the evaluation of the patients; the need arises for a comprehensive evaluation of the lipoylation, respiration state, the level of copper in the labile form and its export processes.

Tumor-specific regulators can change this vulnerability to metabolism. MELK has been suspected of enhancing the development of liver cancer through the regulation of mitochondria related to DLAT (81), while ATP7B dysfunction disturbs the metabolism of both copper and lipids in the cells that make up epithelial tissue (82). Finally, the combination of DSF/Cu can inhibit S6K1 and c-Myc signaling in models of gastric cancer (83). In these works, it is shown that copper can interact with different metabolic processes, instead of only targeting one.

### 4.4 Copper remodeling of the tumor immune microenvironment

Copper is an essential element that affects both tumor and immune cells. In particular, tumoral copper promotes PD-L1 expression via activation of EGFR and STAT3 signaling, whereas copper chelation inhibits this pathway, promoting PD-L1 degradation and enhancing CD8+ T-cell and NK-cell activity (39). Studies with tumor models indicated a decrease in the levels of myeloid-derived suppressor cells (MDSCs), collagen deposition, and increased infiltration of CD4 T cells associated with copper depletion as a result of TM treatment (40). The abovementioned findings indicate that copper depletion has the potential to reverse some immune-evasion strategies but may also impair normal hematopoiesis and immune function (**Figure 4C**).

Similar to copper overload, it appears that the immune effects of copper are context dependent. Although copper ionophores and copper nanoparticles may exert immunological stress, they may also cause nonspecific tissue damage or provocation of mixed cell death pathways. The overall immune implication depends on tumor type, drug distribution, mechanism of death, antigenicity, background immune infiltration, and the capacity of antigen-presenting cells to respond to that antigen. Consequently, demonstration of increased infiltration of CD8+ T cells after copper treatment cannot be viewed as evidence that cuproptosis drives the immune response without the presence of canonical cuproptosis markers (**Figure 4C**).

Studies on thoracic malignancies have also suggested that cuproptosis-related signatures are linked to tumor immunity and effectiveness of therapy (84). Most of these relationships have so far been derived from either computational studies or preclinical models, and need to be validated through evidence from tissues demonstrating cuproptotic pathway activity.

### 4.5 Cuproptosis as immunogenic cell death

An up-to-date mechanistic analysis discovered bidirectional crosstalk between cuproptosis and antitumor immunity (41). Cuproptotic cancer cells released DAMPs that led to the maturation of dendritic cells and activation of CD8+ T cells. In turn, the activated CD8+ T cells secreted IFN-γ, which activated the STAT1–IRF1 axis to increase FDX1 transcription in tumor cells and improve their sensitivity to cuproptosis (41). Across multiple preclinical models, combining a cuproptosis inducer with anti-PD-L1 therapy cuproptosis inducer and anti-PD-L1 therapy was found to be effective against cancer cells and to allow overcoming certain types of resistance to immunotherapy (41) (**Figure 4D**).

These data substantially strengthen the immunological relevance of cuproptosis, but they remain preclinical. Before translation, studies should confirm the death mechanism in patient tumors, define whether the immune effect is preserved across histologies, and distinguish beneficial local immunogenicity from systemic copper toxicity. Biomarker-guided trials should incorporate paired tumor biopsies, circulating immune measurements, and pharmacodynamic evidence of lipoylated-DLAT aggregation or related pathway engagement.

## 5 Copper-related and cuproptosis-associated biomarkers in cancer

### 5.1 Circulating copper, ceruloplasmin, and copper-to-zinc ratio

Serum Cp levels are increased in patients with several malignancies (85). Total serum copper and Cp are therefore frequently studied, and meta-analyses have found higher serum copper and copper-to-zinc ratios in breast and lung cancer than in control populations (86, 87). In a cohort of 583 patients with breast cancer, the highest quartile of the serum copper-to-zinc ratio was associated with worse 10-year survival after multivariable adjustment (88). These findings support further investigation but do not establish a universal screening test.

There are important limitations. Cp is a protein involved in inflammation, and total copper concentrations in serum change in response to Cp changes, meaning both compounds are affected by inflammation, liver function and other factors. In addition, results differ depending on the type of tumor as well. According to a systematic review and meta-analysis performed in 2025, no significant difference was detected between patients suffering from colorectal cancer and control individuals in terms of copper concentrations in the blood or in the tissues (89). It is also important to note that pre-analytical stages of sample processing influence copper concentration in the sample (90). Therefore, it can be concluded that the data obtained should be regarded as non-specific biomarkers of host-tumor effect if assessed without a proper clinical setting.

### 5.2 Tissue and molecular biomarkers

There are likely various tissue biomarkers for copper, such as copper levels either in amount or in where the copper is located, CTR1, ATP7A, ATP7B, ATOX1, FDX1, LIAS, LIPT1, lipoylation status of DLAT, metallothioneins, pathways associated with GSH, and the involvement of OXPHOS processes (6, 24, 26, 35, 36, 49, 51, 91). For cuproptosis evidence of mechanism of how things work should be included when studying the appropriate panel and should not rely solely on gene expression. FDX1 expression gives an indication of a permissive environment, but it is necessary to detect the lipoylation of the protein, aggregation of DLAT, loss of Fe-S-proteins, involvement of respiratory chain, and availability of copper in a tumor.

Several transcriptomic investigations have suggested the utilization of cuproptosis-related prognostic or immune-response signatures in breast carcinoma (92, 93), bladder carcinoma (94), lung carcinoma (95), papillary renal-cell carcinoma (96), and clear-cell renal-cell carcinoma (57). Even though they could help formulate new hypotheses, most of these models operate in parallel, rely on bulk RNA profiles, and fail to provide clear evidence for cuproptosis or the specific mechanisms of copper toxicity. Therefore, it would be fair to call them exploratory signatures rather than validated cuproptosis biomarkers.

Copper-transport proteins can also help predict how patients will respond to various treatments. Patients with increased amounts of CTR1 have better chances of taking up platinum; however, ATP7A and ATP7B reduce the amount of platinum in the cell and provoke resistance (27–30). The level of CTR2 can also influence accumulation of cisplatin and carboplatin and cytotoxic activity in preclinical trials (31). Some exploratory preclinical and clinical studies have reported data linking higher blood copper levels to lack of chemotherapy response (97), but causality has not been established because other factors may contribute. Therefore, biomarker analyses should use pretreatment samples; all stages of trace-element handling should be standardized, and cut-off values for human studies should be predefined.

### 5.3 Predictive biomarkers for copper-targeted treatment

The potential predictive variables depend on the chosen treatment strategy. For tumors with high copper dependence, high CTR1 expression, and metastatic subpopulations that rely on OXPHOS, copper-depletion therapy may be promising (19, 21, 22, 40, 52, 98). Potential predictors of response to copper-overload therapy include high FDX1 levels, intact LIAS/LIPT1-dependent protein lipoylation, active OXPHOS, and limited copper-buffering capacity through GSH or metallothioneins (6, 35, 36, 49, 51). Baseline lactate dehydrogenase (LDH) was associated with outcome as an exploratory subgroup marker in the ES trial, but requires prospective validation (58).

## 6 Cuproptosis in the landscape of regulated cell death

Naming conventions for regulated cell death should be based on fundamental molecular mechanisms instead of morphology or any single marker of injury (53). Though cuproptosis shares features such as mitochondrial dysfunction, oxidative stress, and proteotoxicity with other forms of cell death, the fact that copper forms the basis of cuproptosis through mitochondrial lipoylation and aggregation of lipoylated proteins serves to differentiate it from apoptosis, ferroptosis, necroptosis, and pyroptosis (6, 53). However, mixed phenotypes remain prevalent in many cases, especially with the use of ionophores, DSF/copper, radiation, and materials on the nanoscale.

### 6.1 Cuproptosis versus apoptosis

Apoptosis generally requires caspases and is defined by phenomena like mitochondrial outer-membrane permeabilization or death-receptor signaling, shrinkage of cells, nuclear fragmentation, and limited disruption of cellular membranes (53). Copper overload can induce apoptosis through ROS and mitochondrial damage (50, 62, 63), although the role of caspases does not indicate cuproptosis. However, canonical cuproptosis can occur when some apoptosis inhibitors are still acting (6). It is therefore necessary to use method-specific rescue strategies and molecular markers instead of drawing conclusions about pathways based on observed morphological changes.

### 6.2 Cuproptosis versus ferroptosis

Ferroptosis is an iron-dependent killing program which takes place due to the phospholipid peroxidation and is inhibited by the mechanisms including, as an example, glutathione peroxidase 4 (GPX4) and the GSH (99). Ferroptosis and cuproptosis are both metal-related and can be influenced by GSH; however, ferroptosis is defined by lipid peroxidation, whereas cuproptosis is not and cannot be rescued by conventional ferroptosis inhibitors (6). Nanoparticles that contain copper might work to induce both ferroptosis and cuproptosis simultaneously but must be shown as such separately.

### 6.3 Cuproptosis versus necroptosis and pyroptosis

Necroptosis is executed through RIPK3-dependent phosphorylation and oligomerization of mixed lineage kinase domain-like protein (MLKL), which disrupts the plasma membrane (53, 100). Pyroptosis is executed by gasdermin pores, commonly after inflammatory-caspase activation, and is associated with inflammatory cytokine release (101). Cuproptosis lacks a defined MLKL- or gasdermin-dependent membrane execution module. Nanotherapeutics that combine cuproptosis with pyroptosis can increase immunogenicity, but the term cuproptosis should not be used as a substitute for any lytic copper-associated death (102).

## 7 Therapeutic strategies targeting copper homeostasis and cuproptosis

Classification and application of copper ion-binding compounds have attracted attention in antitumor research, although the field remains at an early stage. These compounds mainly include copper ionophores and copper chelators (103, 104). The former increase intracellular or mitochondrial copper, whereas the latter reduce copper bioavailability. Both are useful pharmacological tools, but limited target selectivity and systemic metal toxicity remain important barriers to clinical translation.

### 7.1 Copper ionophores

Disulfiram, a dithiocarbamate drug approved by the FDA for alcohol dependence, is metabolized to diethyldithiocarbamate, which forms copper complexes. DSF/Cu can induce ROS-dependent apoptosis, proteotoxic pressure mechanism and disrupt NPL4-p97 (62, 65, 105). Certain experimental models might reveal a similarity to cuproptosis, requiring evidence to show whether lipoylation and DLAT aggregation are implicated. Clinical activity in unselected glioblastoma (GBM) has been limited, and a randomized trial showed increased toxicity (106–109). These outcomes highlight the challenges of clinical translation. Copper ionophores may nevertheless have promise in selected tumors by increasing intracellular or mitochondrial copper and overwhelming cellular buffering capacity. ES is able to complex with Cu(II), move to the mitochondria, and release copper via FDX1-dependent and independent mechanisms (34, 110). The capability of this compound to stimulate the process of cuproptosis is greatest in cells with elevated mitochondrial respiration processes and sufficient lipoylation (6, 34). The results of the phase III trial achieved no progress in treating the stage IV melanoma cases in the general population and indicated a death imbalance in patients with high LDH level (58). Such clinical outcomes provide the argument for biomarker-guided therapies rather than unselected use.

Dithiocarbamates consist of a diverse group of ligands that have many types of metal-binding and biological activities (111). Copper-bis(thiosemicarbazone) derivatives are also known to have cytotoxic potential that may manifest in a manner different from that of cisplatin and that also may not be completely reversed by metabolic interventions, as shown in esophageal squamous-cell carcinoma models (112). It is better to categorize these compounds by their intracellular copper management and ability to induce cell death rather than grouping them mistakenly as cuproptosis inducers.

### 7.2 Copper chelators

Copper chelators bind Cu(I) or Cu(II), which reduces copper availability. The goal of copper-depletion methods in oncology is to inhibit copper-dependent signaling, angiogenesis, matrix remodeling, or mitochondrial respiration. D-penicillamine and trientine are known treatments for Wilson disease while TM is still in the research phase; all three agents remain investigational in oncology (113–116). TM can reduce bioavailable copper and Cp as well as cause effects on endothelial progenitor cells and collagen remodeling (40, 52, 71, 114). The greatest challenge is depleting tumor copper without causing adverse effects such as anemia, neutropenia, neurological effects, impaired wound healing or trace-element imbalance.

Along with conventional chelators, new tumor-targeted chelators are under development. A preclinical colorectal-cancer study showed growth suppression with a new copper chelator (117), but efficacy, selectivity, and pharmacokinetics and long-term effects on trace elements must be confirmed by independent studies before being translated into clinical practice.

Chelation could change levels of sensitivity to platinum medications through competition with copper transport proteins or changes in their trafficking. Chelation makes logical therapeutic combinations possible, but it can also decrease the absorption of platinum if there is CTR1 downregulation or redistribution. Treatment design should therefore specify whether the target is circulating copper, tumor copper, mitochondrial copper, a copper-dependent enzyme, or a copper transporter, as these are not equivalent therapeutic objectives.

### 7.3 Copper-based nanotherapeutics and targeted delivery

Nanotherapeutics seek to increase tumor exposure while limiting systemic copper toxicity. Preclinical platforms include stimulus-responsive copper-complex nanoparticles that release Cu(I), glutathione-scavenging celastrol-copper nanoparticles, elesclomol-loaded copper-oxide systems, and ROS-responsive nanoparticles that co-deliver ES and copper (118–121). These platforms have reported DLAT aggregation, immune activation, and improved activity with immune-checkpoint blockade in animal models. Self-assembled copper-chlorogenic-acid nanoparticles have additionally been designed to combine cuproptosis and pyroptosis (102).

The benefits of this new technology are theoretical rather than clinically proven. While nanoparticles can enhance solubility, circulation time, subcellular delivery and trigger-responsive release of the drug, they also bring additional parameters such as batch reproducibility, effects of protein corona, reticuloendothelial uptake, long-term metal persistence, off-target inflammation and scaling-up. Most of the studies that were done to date used small animal model and the efficiency of passive delivery of the nanoparticles may not be achieved in humans. Moreover, it is necessary to conduct studies of distribution of copper in the body, prove cuproptosis *in vivo*, compare it with the free drug and conduct toxicological studies before recognizing it as clinically applicable.

### 7.4 Combination with chemotherapy, radiotherapy, and immunotherapy

Copper modulation may act through several mechanisms/pathways—copper depletion can affect angiogenesis, matrix remodeling processes, OXPHOS, and particular resistance programs, while copper overload can increase mitochondrial stress and overwhelm cellular repair capacity. Studies conducted with nanoparticles have shown the combination of cuproptosis with radiotherapy (RT), ferroptosis, pyroptosis, or chemotherapy; the difference between the synergistic effect and nonspecific additive toxicity must be made (102, 118–121). Studies conducted with the combination must include dose matrices, rescue experiments, and normal tissue controls.

Currently, preclinical evidence supports a rationale for combining copper chelation with immune-checkpoint blockade. Indeed, copper chelation has been shown to downregulate PD-L1 expression, as well as modulate tumor immune infiltrate in proper experimental conditions (39, 40). There is evidence that cuproptosis has immunogenic effect and induces positive feedback on the activity of CD8+ T cells (41). However, the immune modulatory effect might vary in different tumors. Thus, it is necessary to use pharmacodynamic endpoints related to the immune and cuproptosis effects in clinical studies, rather than assume that copper-based solutions will always improve efficacy of checkpoint inhibition in cancer therapy.

## 8 Clinical perspectives

### 8.1 Copper dysregulation in cancer patients

Clinical and observational studies have reported alterations in serum or tissue copper levels during the course of cancer development. Nevertheless, the results are inconsistent. More specifically, elevated serum copper and copper-to-zinc ratios were established in meta-analyses on breast and lung carcinoma (86, 87). The increased copper-to-zinc ratio predicted poor prognosis in breast cancer patients (88). High serum copper concentration was also reported in patients unresponsive to chemotherapy in exploratory studies (97). On the contrary, systematic research on colon cancer did not reveal any significant differences in blood and tissue copper levels (89, 90).

These findings do not support making decisions related to the usage of serum copper levels in screening or treatment. The studies analyzing the data must report on total copper, exchangeable copper, ceruloplasmin-bound copper and tumor soluble copper; their methods of specimen analyzing and processing must be accounted for, too, as well as any influencing factors which are inflammatory, nutritional or hormonal. Copper measurements taken from serum and tumor might present more useful information than just the measurements made separately from any of the items. Also, spatially resolved methods might be used in order to measure the copper distribution in the tumor, since the level of copper is not uniformly distributed in the tumor tissues.

### 8.2 Completed and ongoing clinical trials

Evidence from clinical studies is still inconclusive. In the third phase of the SYMMETRY trial, a total of 651 individuals suffering from stage IV melanoma were administered paclitaxel alone or in combination with ES. Adding ES to treatment regimens did not result in changes in progression-free survival (PFS) in the general population. However, an initial survival analysis showed a death imbalance favoring paclitaxel, predominantly among patients with high baseline LDH. There was a signal regarding PFS rates in a prospectively defined subgroup with normal LDH, but it is still worth looking into (58).

DSF/copper has produced similarly cautionary results. A multicenter phase II study in recurrent temozolomide-resistant GBM reported no objective responses and limited clinical benefit (107). In a randomized phase II/III trial of 88 patients with recurrent GBM, adding DSF/copper to alkylating chemotherapy did not improve survival and increased grade 3 or higher and serious adverse events (106). Two early-phase studies have evaluated DSF/copper in newly diagnosed GBM: the phase I/II study NCT02715609 was completed with 35 participants and has registry results posted, whereas the phase II study NCT03363659 was terminated early at the investigator’s decision after enrolling 15 participants (108, 109). Consequently, the available evidence is insufficient to support the routine clinical use of DSF/copper in GBM.

Evidence is available to show that copper depletion is achievable and provides a pharmacodynamic effect. In a phase II study on breast cancer, it was found that TM was able to deplete copper, and reduce circulating progenitor endothelial cells. The main toxicity of the drug was found to be hematological toxicity at grade 3/4 (71). In a phase II study on GBM where hypocupremia was achieved with the use of penicillamine, patients did not have better survival compared to historical controls (122). TM is currently being evaluated with capecitabine, with or without pembrolizumab, in an ongoing recruiting phase 1b/randomized phase 2 trial of high-risk residual TNBC (NCT06134375); efficacy data are not yet available (123).

### 8.3 Tumor selectivity and patient stratification

A higher average copper concentration in cancer does not imply selectivity for tumor tissue. Healthy organs such as liver, brain, heart, bone marrow, and peripheral nerves rely on strict copper metabolic regulation. Efficient clinical-level approaches need to rely on differential reliance on copper rather than copper concentration per se. Candidate stratification variables include OXPHOS dependence, FDX1 and lipoylation status, levels of labile copper in a tumor, expression and location of CTR1, ATP7A, and ATP7B, metallothionein, GSH pathways, hypoxia and level of LDH (6, 49, 51, 58).

In the case of copper-overload treatment, paired pretreatment and on-treatment biopsies are needed to confirm pathway engagement in the tumoral tissue. For copper depletion, the focus should be on pharmacodynamic targets such as the amount of exchangeable copper or Cp in addition to tumoral marks of the pathway, namely the activity of complex IV, LOX-related matrix markers, or signs of immune remodeling. Endpoints should be prespecified rather than explored *post hoc*.

### 8.4 Mechanisms of therapeutic resistance

Cuproptosis resistance can be developed by reduced copper intake, better export of copper, higher levels of metallothionein or GSH, loss of FDX1 or lipoylation enzymes, lower levels of DLAT, and changes in metabolic activity (6, 34–36, 49, 51). Hypoxia is very important because HIF-1α can mean a decrease in the availability of copper substances and the storage of copper at the same time (51). It seems that gained resistance can include both metal management and metabolic flexibility.

Resistance mechanisms depend on the treatment direction. Resistance to copper depletion may involve increased copper uptake or mobilization from existing stores and alternative angiogenic pathways, whereas resistance to copper overload/cuproptosis may involve compensatory glycolysis or selection of tumor cells that depend less on copper-sensitive mitochondrial metabolism. These possibilities support serial pharmacodynamic monitoring rather than dose selection based solely on body weight.

### 8.5 Copper toxicity and clinical monitoring

Copper overload leads to damage in the liver, nerve tissues, mitochondria, hematologic toxicity, and symptoms affecting the full gastrointestinal system (8). Overexposure or excessive copper depletion from the body through the use of chelators can result in hematological toxicity, such as anemia, neutropenia, or other cytopenias; besides, there are effects associated with particular chelator drugs that should be studied (71, 113, 115, 116, 122, 124). Use of nanoparticles implies an additional risk of infusion reactions, organ accumulation, and side effects specific to drug carriers. Trials should monitor complete blood counts, liver dysfunction, kidney function, neurologic symptoms, the Cp or exchangeable copper, iron and zinc status, and certain cardiac or immune functions related to a particular treatment.

### 8.6 Copper transporters as determinants of platinum-drug response

The machinery responsible for homeostasis of copper in cells manages their intake and excretion, and at the same time is the mechanism used in transmembrane crossing in regard to certain metal-containing anticancer medications. CTR1 is involved in the mechanism of cellular uptake of such drugs as cisplatin, carboplatin and oxaliplatin with high levels of CTR1 expression correlating with higher levels of platinum observed (27, 28). ATP7A and ATP7B contribute to sequestration, efflux, and platinum resistance (29, 30). Furthermore, CTR2 is a key element in the process of accumulation and toxicity of platinum (31).

## 9 Future perspectives

The foremost objective is defining reproducible criteria for cuproptosis both *in vivo* and in patients. The approach should involve copper dependence and measurements of protein lipoylation, DLAT oligomerization/aggregation, Fe-S proteins, mitochondrial respiratory state, and genetic or pharmacological rescue. Only one cuproptosis-related gene signature obtained from bulk transcriptomics analysis is not enough to confirm cell death. Spatial and single-cell studies are vital because the distribution of copper, OXPHOS dependence, hypoxia, and immune infiltration differ within the same tumor.

Biomarker development should proceed alongside drug development. Prospective trials should standardize copper specimen handling, distinguish total from exchangeable or labile copper, and validate tissue assays for FDX1, lipoylated DLAT, metallothioneins, and copper transporters. Functional ex vivo testing may be valuable for determining whether a tumor is more vulnerable to copper depletion or copper overload. Clinical endpoints should be linked to prespecified pharmacodynamic evidence rather than *post hoc* correlations.

Targeted delivery is likely to be vital. Nanotherapeutics of the future must demonstrate active targeting of either tumors or organelles, be manufactured at scale, show quantitative biodistribution, characterization of chronic toxicity, and superiority to a non-targeted drug. The choice of combinations should stem from clearly defined mechanisms of resistance: use of HIF inhibitors for hypoxic cuproptosis resistance, modulation of GSH or metallothionein for copper buffering, targeting transporters for copper efflux, and the immune checkpoint blockade (41, 49, 51).

Lastly, clinical translation necessitates careful analysis of negative research. The unsuccessful studies on ES and DSF/copper are not detrimental for the field of oncology focused on copper, however they indicate that copper dependency does not automatically lead to successful clinical trials without proper pharmacokinetics analysis, tumor targeting and relevant selection of patients (58, 106, 107). Early-phase studies should therefore use biomarker-enriched, mechanism-focused designs before larger trials.

## 10 Conclusions

Copper serves as a “double-edged sword” in tumor biology. As an essential cofactor, it promotes mitochondrial respiration, antioxidant response, oncogenic signaling, extracellular matrix remodeling, angiogenesis, and immune regulation. In excess, mitochondrial copper can bind to lipoylated proteins and promote their aggregation, leading to loss of Fe-S proteins, proteotoxic stress, and cuproptotic cell death. FDX1 connects mitochondrial copper redox biology with LIAS-dependent lipoylation, while TCA-cycle and OXPHOS dependence influence susceptibility to cuproptosis (6, 34–36).

To conclude, cuproptosis, as a newly discovered process of regulated cell death, still lacks great clarity at the molecular level, and many disease biomarkers for its discovery remain uncharacterized. The depletion of copper can block the copper-dependent signaling system, metastasis, angiogenesis and immune evasion. Copper ionophores and target systems of copper-overload could cause cuproptosis to occur in cells. However, tumor selectivity and immunologic efficacy remain context dependent and several clinical trials have yielded inconsistent or negative efficacy results (58, 71, 106, 107, 122). Therefore, attaining success requires defining mechanisms, identifying biomarkers, targeting the tumor and monitoring pharmacodynamics. In conclusion, copper-related precision oncology looks promising but has to be used together with defined biomarkers.

## Glossary

ACSL4: acyl-CoA synthetase long-chain family member 4
AKT: protein kinase B
ATOX1: antioxidant 1 copper chaperone
CCS: copper chaperone for superoxide dismutase
COX17: cytochrome c oxidase copper chaperone 17
Cp: ceruloplasmin
CTR1: copper transporter 1 (SLC31A1)
DAMPs: damage-associated molecular patterns
DLAT: dihydrolipoamide S-acetyltransferase
DLD: dihydrolipoamide dehydrogenase
DSF: disulfiram
ECM: extracellular matrix
EPC: endothelial progenitor cell
ES: elesclomol
FDX1: ferredoxin 1
Fe-S: iron-sulfur
GBM: glioblastoma
GCSH: glycine cleavage system protein H
GPX4: glutathione peroxidase 4
GSH: glutathione
HIF: hypoxia-inducible factor
IFN-γ: interferon-γ
IRF1: interferon regulatory factor 1
LDH: lactate dehydrogenase
LIAS: lipoic acid synthase
LIPT1: lipoyltransferase 1
LOX/LOXL: lysyl oxidase/lysyl oxidase-like
MDSCs: myeloid-derived suppressor cells
MLKL: mixed lineage kinase domain-like protein
NK: natural killer
NPL4: nuclear protein localization protein 4
ORR: overall response rate
OS: overall survival
OXPHOS: oxidative phosphorylation
PD-L1: programmed death ligand 1
PDK1 (AKT pathway): 3-phosphoinositide-dependent protein kinase 1
PDK1/3 (TCA/Metabolism): pyruvate dehydrogenase kinase 1/3
PFS: progression-free survival
RIPK3: receptor-interacting serine/threonine-protein kinase 3
ROS: reactive oxygen species
RT: radiotherapy
SLC31A1: solute carrier family 31 member 1 (CTR1)
TCA: tricarboxylic acid
TGN: trans-Golgi network
TM: tetrathiomolybdate
TMZ: temozolomide
TNBC: triple-negative breast cancer
VCP: valosin-containing protein (p97)
